# Tumor-Associated Macrophage Promotes the Survival of Cancer Cells upon Docetaxel Chemotherapy via the CSF1/CSF1R–CXCL12/CXCR4 Axis in Castration-Resistant Prostate Cancer

**DOI:** 10.3390/genes12050773

**Published:** 2021-05-19

**Authors:** Wei Guan, Fan Li, Zhenyu Zhao, Zongbiao Zhang, Junhui Hu, Yan Zhang

**Affiliations:** 1 Department of Urology and Institute of Urology, Tongji Hospital, Tongji Medical College, Huazhong University of Science and Technology, Wuhan 430030, China; fanli117@hotmail.com (F.L.); zhaozhenyutjm@163.com (Z.Z.); zzb070@126.com (Z.Z.); zhangyanhust@163.com (Y.Z.); 2 Department of Molecular and Medical Pharmacology, David Geffen School of Medicine, University of California at Los Angeles, Los Angeles, CA 90095, USA; junhuihu@mednet.ucla.edu

**Keywords:** castration-resistant prostate cancer, tumor-associated macrophage, CSF-1, CXCR4, CXCL12

## Abstract

Castration-resistant prostate cancer (CRPC) is an advanced stage of prostate cancer that can progress rapidly even in patients treated with castration. Previously, we found that tumor-associated macrophages (TAM) can be recruited by CSF-1 secreted by docetaxel-treated prostate cancer cells and promote the survival of cancer cells in response to chemotherapy. The inhibition of CSF-1R can impede this effect and significantly prolong survival in xenograft mice. However, the actual mechanism of how TAM improves cancer cell survival still remains elusive and controversial. Here, for the first time, we found that the enhanced survival of cancer cells achieved by TAM was mainly mediated by CXCR4 activation from the increased secretion of CXCL12 from CSF-1 activated TAM. This finding helps to clarify the mechanism of chemoresistance for second-line chemotherapy using docetaxel, facilitating the development of novel drugs to overcome immune tolerance in castration-resistant prostate cancer.

## 1. Introduction

Prostate cancer is the most common cancer in men and the leading cause of mortality caused by cancer in men after lung and bronchus in the United States in 2021 [1]. A total of 248,530 new cases and 34,130 deaths have been estimated to occur within 2021 [1]. However, nearly 100% of all patients who are in the early stages with a localized or regional primary tumor can be cured, and around 98% of patients overall may receive favorable 5-year survival prognoses [1]. Despite this, patients found with distant metastases, especially metastases to the bone, only have a 30% chance of survival after 5 years with the treatment regimens currently available [1].

Androgen deprivation therapy (ADT) was developed more than 75 years ago and has been the standard of care as first-line therapy. It represents a large category of treatment plans, from surgical orchidectomy initially, to blockage of the androgen receptor (AR) and androgen synthesis pathways through abiraterone and enzalutamide. However, some of the patients in the advanced stages of cancer who receive ADT progress to become androgen-independent, in a condition known as castration-resistant prostate cancer (CRPC). Furthermore, the second-line therapy including docetaxel and prednisone may also fail to control the aggressiveness of CRPC in the late stages. Immunotherapy is one of the most important achievements in cancer treatment and has received the most attention and support. Importantly, Vincenza Conteduca et al. reported that metastatic CRPC patients treated with enzalutamide with a persistent neutrophil to lymphocyte ratio (NLR) > 3 were found to have a poorer prognosis than those with NLR ≤ 3 [2]. Matteo Santoni et al. also reviewed the critical role of lymphocytes, macrophages, and other inflammatory cells in the secretion and regulation of CXCL12 in tumors such as prostate cancer [3]. All these studies implicate the intricate but pivotal involvement of immune cells in prostate cancer and explain the urgent need for further mechanistic investigation in this field.

Against this backdrop, we previously proposed a compound treatment regimen incorporating both the colony-stimulating factor 1 (CSF-1) receptor antagonist PLX 3397, or pexidartinib, and docetaxel. This compound therapy prevents the recruitment of monocytes from circulation to the cancer tissue and diminishes the transition of tumor-associated macrophages (TAMs) from M1 to M2 subtype, which is acknowledged as a key component of immune tolerance in the tumor microenvironment. However, the mechanism of TAMs promoting cancer cell survival remains elusive. In this study, we show that CXCL12 from TAMs exerts a paracrine effect on cancer cells via CXCR-4 and protects cancer cells from damage caused by docetaxel.

## 2. Materials and Methods

### 2.1. The Cell Culture, Reagents, and Generation of Genetic Engineered Cell Lines

MyC-CaP (ATCC, Manassas, VA, USA) and RAW264.7 (RAW) (National Collection of Authenticated Cell Cultures at Shanghai, China) were cultured in DMEM media supplemented with 10% charcoal stripped fetal bovine serum (FBS), which mimics the low-androgen condition in castration-resistant prostate cancer, and 1× Penicillin–Streptomycin. PC-3 (National Collection of Authenticated Cell Cultures at Shanghai, China) and C4-2 (ATCC, Manassas, VA, USA) were cultured in RPMI 1640 media supplemented with 10% FBS and 1× Penicillin–Streptomycin. All cells were cultured in 37 °C, 5% CO_2_ humidified cell incubators. Pexidartinib was purchased from Selleck (Cat#S7818, Shanghai, China). The overexpression plasmid of murine CXCL12 was developed through Gibson assembly of the PCR product from the forward primer 5′-CGCCAGAACACAGGACCGGTTCTAGAATGGACGCCAAGGTCGTC-3′ and the reverse primer 5′-CATCGTCTTTGTAATCCATCTCGAGCTTGTTTAAAGCTTTCTCCAGGTAC-3′, and the double digested plasmid lentiCRISPRv2 hygro from Addgene (Cat#98291, Watertown, MA, USA) was developed with Gibson assembly mastermix (Cat#E2611S, NEB Biolabs, Ipswich, MA, USA). The ELISA kit used to examine the CXCL12 from RAW cells was purchased from R&D Systems (Cat#MCX120, Minneapolis, MN, USA), and ELISA was undertaken in accordance with the manufacturer’s protocol.

### 2.2. Cell Proliferation Assay

Cell proliferation was analyzed using MTS assay from Promega (Cat#G3580, Dane County, WI, USA). On day 0, cells in the log phase were counted and seeded in a 24-well plate with a flat bottom and transwell chambers at 10,000/well and chamber. The absorbance of wells in each group was examined after 48 h, and incubation time with MTS reagent at 1 h. Absorbance was measured with a Multiskan MK3 microplate reader (Thermo, Waltham, MA, USA). The docetaxel IC50s for prostate cancer cells were determined previously in our work [4] and added to PC-3 (30 nM) and C4-2 (2 nM) cells for 48 h. The PLX-3397 working concentration was also previously determined to be 2 μM [4], and a control group with Dimethyl sulfoxide (DMSO) at the same amount was included. The cell treatment regimens for the colony formation assay and transwell assay were kept the same, and the culture volume was accordingly amplified to meet the cell number requirement for each assay.

### 2.3. Colony Formation Assay

Colony formation assay is another effective method to evaluate the growth potential of cells by assessing the colony size and numbers, within the same period of 2 weeks in our experiment. Cells in each group were cultured in the log phase, treated as previously mentioned in the Results section for 48 h and counted to be seeded in a 6-well plate with 1000/well. Cell culture media were changed every 2–3 days until the endpoint of 2 weeks was reached. All cells in each well were fixed with 0.4% paraformaldehyde and stained with 0.01% crystal violet for 15 min, followed by tap water flush for 5 min. All wells were photographed. Colonies were counted using ImageJ 1.53C (downloaded from https://imagej.nih.gov/ij/, accessed on 10 January 2020) and measured for statistical analysis.

### 2.4. Western Blot and Quantitative Real-Time Polymerase Chain Reaction (qRT-PCR)

Western blotting was undertaken as described in Xin-Wei Diao et al. [5]. The antibodies used in this study include anti-CXCR4 (Cat#NB100-74396SS, Novus Bio., Littleton, CO, USA) and anti-β-Actin (Cat#sc-47778, Santa Cruz Biotech, Dallas, TX, USA).

For qRT-PCR, the total RNA of the cell was extracted via the traditional phenol–chloroform method and reverse-transcribed using a PrimeScript RT Reagent Kit from Takara, Japan (Cat# RR037B). Then, cDNA was quantified via PCR with a 7500 Fast Real-Time PCR System from Thermofisher, USA (Cat#4351107) using SYBR Premix Ex Taq from Takara, Japan (Cat#RR420). The forward primer for CXCL12 is 5′-TGCATCAGTGACGGTAAACCA-3′, and the reverse primer is 5′-TTCTTCAGCCGTGCAACAATC-3′. The forward primer for CXCR4 is 5′-ACTACACCGAGGAAATGGGCT-3′, and the reverse primer is 5′-CCCACAATGCCAGTTAAGAAGA-3′.

### 2.5. Statistical Analysis

All experiments were repeated three times, and data are presented as mean ± SEM. Student’s *t*-test was used for comparison between two groups, while two-way ANOVA was used for comparisons between multiple groups.

## 3. Results

### 3.1. Docetaxel Treatment Induces Upregulation of CXCR4 in Prostate Cancer Cells, and Artificial Stimulation of CSF-1 Increases CXCL12 Production in Macrophages

**Figure 1:**
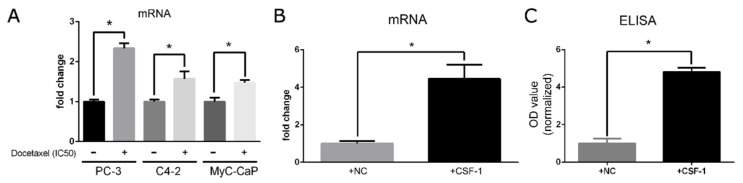
Docetaxel treatment for 48 h induces the upregulation of CXCR4 in prostate cancer cells and CXCL12 in macrophages. (**A**) CXCR4 was upregulated upon treatment with docetaxel in PC-3, C4-2, and MyC-CaP cells via qRT-PCR. (**B**) CXCL12 was increased when RAW cells were stimulated by CSF-1 as examined via qRT-PCR. (**C**) ELISA confirmed the increased secretion of CXCL12 when stimulated by CSF-1 in RAW cells. All experiments were repeated three times. *: *p* < 0.05.

To explore the impact of the second-line chemotherapy drug docetaxel on cancer cells and macrophages, we interrogated the mRNA changes induced by CXCR4 on cancer cells. As shown in Figure 1A, CXCR4 was upregulated following docetaxel treatment in prostate cancer cell lines including PC-3, C4-2, and MyC-CaP. Since CXCL12 is a classical ligand of CXCR4 [5] and CSF-1/CSF-1R crosstalk from prostate cancer cells to recruited macrophages was previously identified, we proposed that the feedback crosstalk from macrophages to cancer cells is partly mediated by CXCL12/CXCR4. Consequently, CSF-1 was artificially supplemented at 100 ng/mL to RAW cells for 48 h, and we examined the mRNA from RAW cells via qRT-PCR and supernatant proteins via ELISA. Both mRNA and protein levels validated our hypothesis that CSF-1 can induce the production of CXCL12 in tumor-associated macrophages, as shown in Figure 1B,C.

### 3.2. CSF-1R Inhibitor Pexidartinib Can Negate the Pro-Survival Effect of Tumor-Associated Macrophages (TAM)

**Figure 2:**
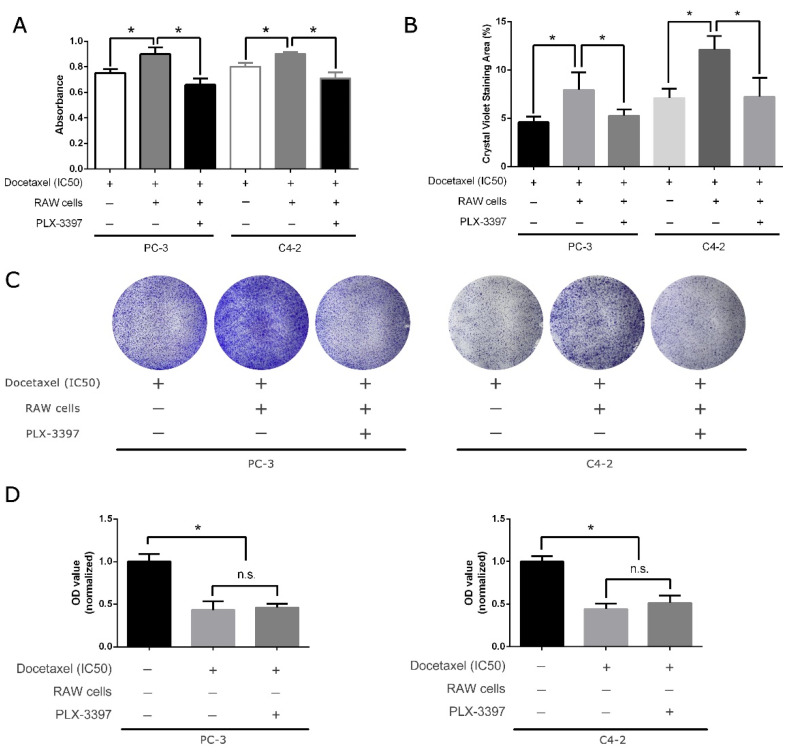
CSF-1R inhibitor pexidartinib can negate the pro-survival effect caused by tumor-associated macrophages. (**A**) Pexidartinib can restore sensitivity to docetaxel in PC-3 and C4-2 cells in MTS assay. (**B**) In the colony formation assay, pexidartinib was also shown to have an effect on removing protection from RAW cells in PC-3 cells and C4-2 cells. (**C**) Representative images of colony formation assay stained by crystal violet. (**D**) Without RAW cells, pexidartinib alone does not change docetaxel sensitivity in PC-3 and C4-2 cells. All treatment durations were 48 h before cell seeding in the colony formation assay, and experiments were repeated three times. *: *p* < 0.05; n.s.: not significant.

Our previous study reported that intraperitoneal administration of CSF-1R inhibitor pexidartinib can partially restore docetaxel sensitivity in both the subcutaneous xenograft mouse model from MyC-CaP cells and orthotopic model with CWR22Rv1 and significantly attenuate the growth of primary tumor and achieve longer survival in xenograft mice. Here, to cross-validate and confirm this in an in vitro study, we assessed the sensitivity of two human prostate cancer cell lines, PC-3 and C4-2, toward docetaxel with and without pexidartinib. Unsurprisingly, the combined chemotherapy of pexidartinib and docetaxel helps to regain sensitivity toward docetaxel, as reflected in the MTS assay shown in Figure 2A and colony formation assay in Figure 2B,C. Additionally, to further examine the role of RAW cells and pexidartinib, we supplemented the cancer cells’ IC50s with docetaxel and pexidartinib in the absence of RAW cells. No sensitivity changes in docetaxel on cancer cells were observed in comparison to docetaxel alone (Figure 2D). This confirms that pexidartinib can eliminate the pro-survival effect of macrophages in the tumor microenvironment and mimic the effect of macrophage absence.

### 3.3. Knockdown of CXCR4 Can Mimic the Effect Caused by CSF-1R Inhibition

**Figure 3:**
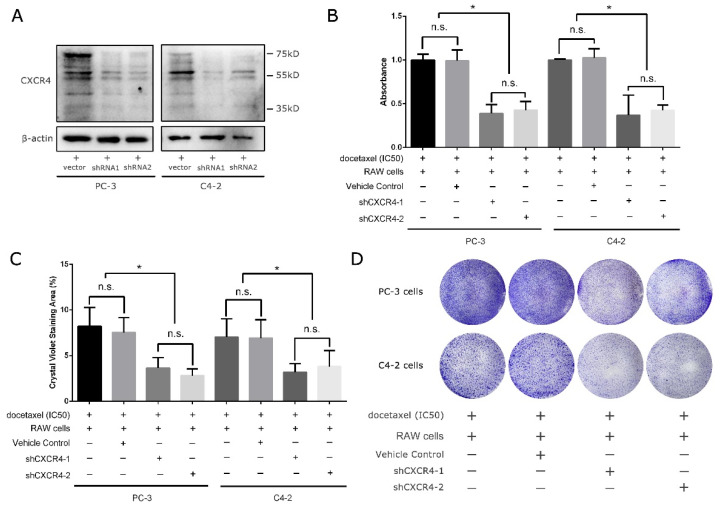
Knockdown of CXCR4 can mimic the effect caused by CSF-1R inhibition. (**A**) Western blotting showed a remarkably downregulated CXCR4 in both PC-3 and C4-2 cells with two shRNAs. (**B**) MTS assay showed that downregulation of CXCR4 in PC-3 and C4-2 cells can mimic the effect caused by CSF-1R inhibition. Additionally, the colony formation assay confirms this finding, as shown in (**C**,**D**). All treatment durations were 48 h before cell seeding in the colony formation assay, and experiments were repeated three times. *: *p* < 0.05; n.s.: not significant.

As previously mentioned, we propose that CSF-1R activated macrophages can have crosstalk with cancer cells via the CXCL12/CXCR4 axis. To examine the role of CXCR4 in response to either docetaxel treatment in the presence of activated macrophages, we knocked down CXCR4 expression in prostate cancer cells including PC-3 and C4-2, with two distinct pairs of short hairpin RNA targeting two different loci in the third exon of human CXCR4, which is shared across five isoforms from the PubMed gene database. Upon lentivirus-mediated stable transfection, as shown in Figure 3A, both shRNA1 and shRNA2 can effectively downregulate the level of CXCR4 in cells PC-3 and C4-2. Then, we cocultured RAW cells with either PC-3 or C4-2 cells in the transwell system, in which RAW cells were in the transwell chamber and cancer cells in the bottom 24-well plate and supplemented with docetaxel in the cancer cells’ IC50s. Interestingly, the downregulation of CXCR4 in these two prostate cancer cells surprisingly mimicked the effect caused by CSF-1R inhibition, as seen in Figure 2, and restored the sensitivity of cancer cells to docetaxel (Figure 3B–D). This supports the theory that CXCR4 in cancer cells might be one of the critical docetaxel-resistance regulators and is potentially involved in CXCL12 (macrophage)/CXCR4 (cancer cell) crosstalk.

### 3.4. Overexpression of CXCL12 in Macrophages Can Negate the Effect Caused by CSF-1R Inhibition from Pexidartinib

In spite of the evidence that CXCR4 may be involved in docetaxel-resistance regulation, a direct link from CXCL12 by macrophage to CXCR4 in the cancer cells cannot be confirmed. With this concern in mind, and considering the fact that CXCR4 is a major receptor of CXCL12, we further tested whether artificial overexpression of ligand CXCL12 in macrophages can prevent the resensitization of cancer cells to docetaxel in the presence of pexidartinib. To this end, we cocultured RAW cells with either PC-3 or C4-2 cells in a transwell system in the presence of both docetaxel and pexidartinib in the cancer cells’ IC50s. Strikingly, and consistent with our hypothesis, the overexpression of CXCL12 in the macrophages can remarkably enhance the survival of cancer cells despite the existence of pexidartinib. Therefore, artificial overexpression of CXCL12 can rescue cancer cells in docetaxel and pexidartinib, which would otherwise be killed by CSF-1R inhibition.

## 4. Discussion

Pexidartinib is a competitive ATP inhibitor of the CSF-1 receptor kinase and was approved by the US Food and Drug Administration (FDA) in 2019 for use in tenosynovial giant cell tumors [4]. Further, it was found to be effective in controlling cancers including prostate cancer [4], ovarian cancer [6], glioblastoma [7], and melanoma [8]. It is interesting that almost all studies found it to be ineffective as a single drug for these diseases, but it was found to be beneficial and to improve prognosis in combined therapy with other regimens. This might due to the adjuvant role of macrophages as “helper cells” in T-cell-mediated cancer cell killing [8] and the formation of a tumor-tolerant microenvironment [9], rather than being directly involved in the cytotoxic process. Even though pexidartinib was reported to function through targets other than CSF-1R, such as cKIT, FMS-like tyrosine kinase 3 (FLT3), and platelet-derived growth factor receptors β (PDGFR-β), no observable differences were found in our previous study when pexidartinib was used alone in a prostate cancer xenograft mouse model [4]. Therefore, in this study, we mainly discussed combined therapy incorporating both docetaxel and pexidartinib. As shown in Figure 2, the combined treatment can effectively prevent the acquired resistance caused by polarized macrophages.

The CXCL12/CXCR-4 axis is one of the most important pathways involved in cancer growth and metastasis. CXCR-4 overexpression is correlated with poor prognosis in a broad spectrum of cancers, including colorectal cancer [10], melanoma [11,12], breast cancer [13], and prostate cancer [14]. Importantly, multiple studies [5,15,16] have reported that CXCL12/CXCR-4 axis activation enhances cancer cell adhesion, migration, and invasion, leading to increased metastasis, and specific blockage via neutralizing antibodies targeting CXCR4 can partly interrupt this process. CXCR4 and CXCL12 can be induced under different conditions. Here, for the first time, we found that they can also be upregulated upon treatment with docetaxel (Figure 1). One possible explanation is the upregulation of various interleukins such as IL-10 induced by docetaxel treatment, as shown previously in our study [4], and the fact that interleukins including IL-10 [17], IL-2 [18], IL-4 [19], and IL-7 [19] are all potent CXCR-4 inducers. Regarding CXCL12′s increase in macrophages, by contrast, it is reported that chemotherapy drugs such as cyclophosphamide and 5-fluorouracil can increase CXCL12 in osteoblasts [20]. It is thus reasonable to infer that it might follow the same paradigm in macrophages, but this remains to be validated.

**Figure 4:**
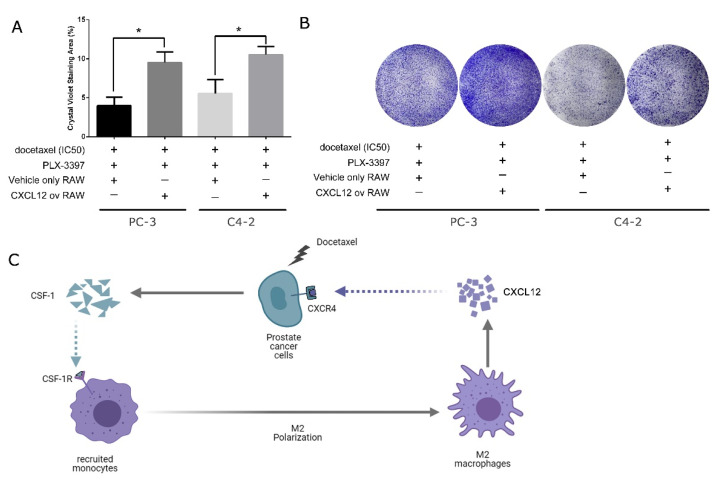
Overexpression of CXCL12 in macrophages can negate the effect caused by CSF-1R inhibition from pexidartinib. (**A**) Upon artificial expression of CXCL12 in RAW cells, RAW cells can prevent the resensitization of CSF-1R inhibition caused by pexidartinib in both PC-3 and C4-2 cells. (**B**) Representative images of colony formation assay stained by crystal violet. (**C**) Diagram describing the crosstalk of polarized macrophages and castration-resistant prostate cancer cells in response to docetaxel treatment. All treatment durations were 48 h before cell seeding in the colony formation assay, and experiments were repeated three times. *: *p* < 0.01; ov: overexpressing.

Interestingly, Urszula M Domanska et al. [21] and Arun Bhardwaj et al. [22] coincidentally reported the counteracting effect between CXCL12/CXCR4 signaling and docetaxel treatment, and inhibition of this axis can significantly sensitize prostate cancer to docetaxel treatment. This further supports our findings regarding the crosstalk between macrophages and castration-resistant prostate cancer cells in response to the second-line therapeutic agent docetaxel, as shown in Figure 4C. Against this backdrop, our study is the first to take macrophages into consideration in docetaxel treatment in the study of the CXCL12/CXCR4 axis and found an alternative therapy comparable to our previously reported regimen, which includes both CSF-1R antagonist and docetaxel (Figure 3 and Figure 4), thus improving our understanding of the crosstalk between macrophages and prostate cancer cells. It is inevitable that disease will eventually progress in CRPC patients, and they will experience relapses even with combined pexidartinib and docetaxel treatment, which was also seen in our prostate cancer xenograft mouse model [4]. Therefore, this study is of great significance as it can serve as a last resort when even pexidartinib fails in CRPC patients and can help to improve overall survival.

## 5. Conclusions

Upon the combined treatment of docetaxel and ADT, prostate cancer cells can recruit and induce the polarization of the tumor-associated macrophages to release CXCL12, which in turn promotes the survival of cancer cells via CXCR4 and mediate the drug resistance.

## Data Availability

Data is contained within the article or supplementary material.
